# Macronutrient-Based Predictive Modelling of Bioconversion Efficiency in Black Soldier Fly Larvae (*Hermetia illucens*) Through Artificial Substrates

**DOI:** 10.3390/insects16010077

**Published:** 2025-01-14

**Authors:** Laurens Broeckx, Lotte Frooninckx, Siebe Berrens, Sarah Goossens, Carmen ter Heide, Ann Wuyts, Mariève Dallaire-Lamontagne, Sabine Van Miert

**Affiliations:** 1 Centre of Expertise Sustainable Biomass and Chemistry, Campus Geel, Thomas More University of Applied Sciences, Kleinhoefstraat 4, 2440 Geel, Belgium; lotte.frooninckx@thomasmore.be (L.F.); siebe.berrens@thomasmore.be (S.B.); sarah.goossens@thomasmore.be (S.G.); carmen.terheide@thomasmore.be (C.t.H.); ann.wuyts@thomasmore.be (A.W.); sabine.vanmiert@thomasmore.be (S.V.M.); 2 Département des Sciences Animales, Faculté des Sciences de L’Agriculture et de L’Alimentation, Université Laval, 2425 rue de l’Agriculture, Québec, QC G1V 0A6, Canada; marieve.dallaire-lamontagne.1@ulaval.ca

**Keywords:** black soldier fly, macronutrients, modelling, bioconversion, artificial substrates

## Abstract

This study explores the optimisation of rearing substrates for black soldier fly larvae (BSFL). First, the ideal dry matter content of substrates was determined, comparing the standard 30% dry matter (DM) with substrates hydrated to their maximum water holding capacity (WHC). Substrates at maximal WHC yielded significantly higher larval survival rates (*p* = 0.0006). Consequently, the WHC approach was adopted for further experiments. Using these hydrated artificial substrates, fractional factorial designs based on central composite and Box–Behnken designs were employed to assess the impact of macronutrient composition on bioconversion efficiency. The results demonstrated significant main, interaction, and quadratic effects on bioconversion efficiency. Validation with real-life substrates of varied protein content, including indigestible feather meal, affirmed the predictive model’s accuracy after accounting for protein source digestibility. This research underscores the importance of optimal hydration and macronutrient composition in enhancing BSFL growth and bioconversion efficiency.

## Simple Summary

As the global population continues to grow, the demand for sustainable protein sources is intensifying. Black soldier fly (BSF) larvae are emerging as a promising solution for reducing food waste while producing high-quality proteins for animal feed. This study focused on optimising the growth of BSF larvae by exploring how their feed’s nutrient content impacts their growth. By testing various feed formulations and developing a predictive model, we created a prediction formula for balancing protein, fat, and carbohydrates to maximise growth and efficiency. These findings provide a roadmap for creating better feed mixes from diverse organic waste streams, helping reduce waste and dependency on traditional protein sources like soy and fishmeal.

## 1. Introduction

Current predictions estimate a world population of 9.7 billion by 2050 [1]. In addition to this increase, prosperity in current developing countries is expected to grow, typified by a change in diet whereby more animal proteins are consumed. In order to produce animal proteins, high feed inputs are necessary. For example, currently 36% of all cultivated crops are used as animal feed [2]. Proteins are an important component in these feeds and are mainly derived from oilseed meal (in particular soy), by-products of biodiesel production and fish [3]. However, since 1970, overfishing has been a major issue and current fisheries exceed the maximum capacity for ensuring a sustainable biological system [4]. This means a further depletion is detrimental for all fish stocks. Moreover, the sustainability of soy production can be questioned regarding the deforestation of rainforests [5]. High demand for soy protein for both human food and livestock feed leads to excessively high production of soy in South-American countries. To meet the soy demands, deforestation (not only by cutting, but also by burning) happens rapidly to increase the area for soy cultivation. Strikingly, since 2021, this has led to the Amazon rainforests, previously known as the lungs of the earth, to become CO_2_ emitters [6,7].

In order to thrive on an overcrowded planet in the long-term, it is important to relieve pressure on the most conventionally used protein sources such as fishmeal and soy and produce proteins sustainably [8]. Insect farming, specifically the production of the larvae of *Hermetia illucens* (Linnaeus), commonly known as the black soldier fly (BSF), has the potential to serve as a crucial component in the agro and feed industry [9]. This is attributed not only to their exceptional bioconversion efficiency but also their remarkable digestion plasticity, which enables their growth using diverse organic side-streams [10,11]. It has already been shown that BSF larvae have a favourable feed conversion ratio, nutritional composition, and amino acid composition [12,13,14]. The dry weight can contain up to 35% fat and 50% protein, with an amino acid profile similar to that of fishmeal [15]. The side-streams that allow BSF rearing include various sources, including manure, waste-streams with high microbial loads, supermarket and domestic food waste, as well as agricultural side-streams that are unsuitable for human and animal consumption and therefore treated as organic waste [10,11]. Studies have shown that BSFs are extremely efficient in converting diverse sources of organic materials into larval biomass [16,17], showing reductions in organic waste up to 84.8% [17,18]. Organic waste constitutes 47% of the global total waste [19]; moreover, roughly one-third of the global food production for human consumption (ca. 1.3 billion tons per year) is lost or wasted [20]. Since BSFs can be used for the reduction of waste streams, as well as the production of high-quality protein, they contribute to increased circularity in our agro and feed industry, relieving the pressure on conventional protein sources [11,15].

Although the BSF industry has made significant progress in recent years, the use of waste or side-streams as a substrate for the production of BSFs remains largely unconsolidated. Moreover, the waste streams used are often already valorised as feed for conventional livestock, thus creating direct competition with this sector [21]. However, the limited knowledge regarding the nutritional requirements of BSFs and the lack of a stable supply chain for waste streams as feed for BSFs, often makes producers reluctant to use them.

This is in sharp contrast to conventional livestock. For pig, cattle, and poultry, we possess detailed knowledge regarding the feed requirements for each developmental stage. This knowledge allows feed companies to successfully valorise side-streams as tailor-made feed for livestock. Even though the insect sector has made big advancements in the last decade, there are several studies that have evaluated growth of BSF larvae on specific waste streams [11,12,22]. Unfortunately, due to large variations in experimental setup and composition of the side-streams (even with similar streams sampled at different time spans or locations), often varying results are obtained [11]. Some of these studies have also attempted to model the nutritional requirements of BSF larvae, but modelling showed varying results due to the complexity of these streams [23,24,25].

In order to optimise BSF production, it is essential to unravel how the substrate macronutrient composition influences larval growth and the larval nutritional composition [23,26]. However, modelling larval growth based on side-streams is demanding due to the variation in nutritional composition, including challenging-to-measure components such as different types of mono- and polysaccharides, and variations in amino acid and fatty acid profiles [11]. Thus, to address these challenges, several studies used artificial substrates to overcome this issue [23,27].

Variations in amino acid profiles have a significant influence on larval growth, while the types of carbohydrates, including short sugars, long sugars, and different starch types, play a pivotal role in altering the digestive processes [28]. Furthermore, diverse fat sources are reflected in larval fatty acid compositions [28]. Notably, the fibre profile, encompassing lignin, cellulose, and hemicellulose contents, as well as the types of hemicelluloses, also significantly impact larval digestive processes [29]. Some fibres can serve as an energy source, whereas others can negatively affect larval growth [29].

Previous research has demonstrated the potential of artificial substrates in modelling the nutritional requirements for larvae. However, in previous studies, little attention was given to the physical composition of the substrates (e.g., adapted substrate dry matter contents based on their water holding capacity (WHC)) [30]. This study takes a step forward from previous research by incorporating WHC methods developed by Frooninckx et al. (2024), utilising artificial substrates and employing a more intricate experimental design. This study aimed to develop a reliable model for accurately predicting larval growth parameters, specifically focusing on the larval mass produced when reared on various side-streams. By examining the effects of feed macronutrient contents on the growth of black soldier fly larvae, we aimed to provide valuable insights into optimising substrate formulations. This approach offers practical benefits for the industry by enabling the blending of nutritionally diverse side-streams to create ideal feed substrates, ultimately enhancing the efficiency and sustainability of BSF production systems.

## 2. Materials and Methods

### 2.1. Preparation of Feed Substrates

For composing artificial substrates, sunflower oil (Vandemoortele, Ghent, Belgium), wheat starch (Tarwezetmeel, Van Beekum specerijen, Harderwijk, The Netherlands), casein (organic casein protein, Ekopura, Hoofddorp, The Netherlands) and cellulose (Alphacel, MP Biomedicals, Santa Ana, CA, USA) were used. Composing artificial substrates, solely on cellulose, starch, casein, and sunflower oil, can potentially lead to deficits in essential micronutrients or substances such as sterols [31]. Therefore, it was decided to use a base amount of chicken start mash (Chicken Start Mash 259, AVEVE, Geel, Belgium) of 8% dry matter (DM) for each different substrate composition. For the optimisation of the substrate dry matter content, potato starch was used instead of wheat starch. However, due to poor digestibility, for later experiments it was replaced with wheat starch. The macronutrient compositions of the ingredients are displayed in Table 1 and were provided by the suppliers of the feed ingredient producers.

The experimental setup was divided into three phases:

**Optimisation of substrate dry matter content (1)**: This phase aimed to determine the optimal dry matter content for the artificial substrates, ensuring larval growth.

Therefore, different hydration levels for a range of artificial substrate compositions were evaluated to identify the hydration condition that maximises performance or suitability for subsequent experiments.

**Development of artificial substrates and prediction models (2)**: This phase involved the creation and testing of artificial substrates. Data from these experiments were used to develop predictive models that can forecast the performance of the substrates under various conditions.

**Validation of the experimental design (3)**: This phase was dedicated to validating the accuracy and reliability of the developed models. This involved testing the models under different conditions with real side-streams to ensure their predictive validity and robustness.

#### 2.1.1. Optimisation of Substrate Dry Matter Content

In the experiment to **optimise the substrate dry matter content**, the design displayed in Table 2 was used. Substrates with the displayed macronutrient contents were hydrated up to (1) a dry matter content of 30% and (2) their maximal water holding capacity (WHC). The amounts of each ingredient added to formulate the diet are displayed based on the dry matter content of the substrate.

WHC was determined by adding 3 g of fresh substrate to a 50 mL falcon and adding an excess amount of water (25 mL). The substrates underwent vortexing for homogenisation and were subsequently left at room temperature for 1 h. Following this, the falcons were subjected to centrifugation at 10,000× *g* for a duration of 30 min. After centrifugation, the water layer was decanted, and the residual liquid was allowed to drain for 30 min. The substrate, along with the bound water, was then reweighed to determine the water holding capacity using the formula provided below [30].

(1) $$WHC\left(\%\right)=\frac{m_{falcon\;tube+substrate\;after\;centrifugation}(g)-m_{falcon\;tube+substrate}(g)}{m_{substrate}}\times 100\%$$

The WHC indicates the amount of water required to fully hydrate the substrate without any free water remaining. For example, a WHC of 150% means that 20 g of substrate would need 30 g of water to achieve optimal hydration.

#### 2.1.2. Development of Artificial Substrates and Prediction Models

**Figure 1:**
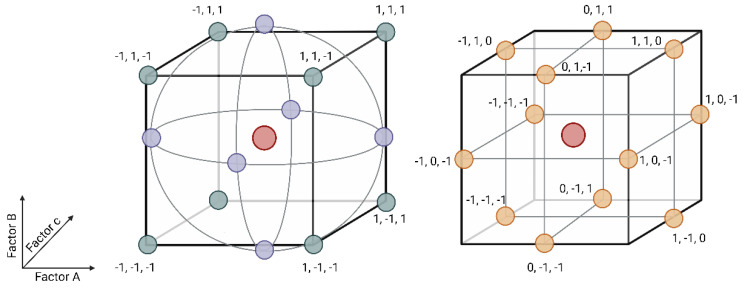
Central Composite design (**left**) and Box–Behnken design (**right**). (Figure was adapted from Breig and Luti [32]).

To formulate diverse feed substrates, a combination of a central composite and a Box–Behnken design methodology [32] was used. Figure 1 illustrates the arrangement of the Central Composite design (on the left) and the Box–Behnken design (on the right). Our aim was to assess the impact of substrate carbohydrate, protein, and fat content on larval bioconversion efficiency; thus, we set boundaries based on established substrates commonly used in black soldier fly (BSF) breeding [11].

The macronutrient contents were categorised as (−α, −1, 0, +1, +α), wherein −1 represented the lower boundary, +1 the upper boundary, and 0 denoted the midpoint. Additionally, axial points (depicted as blue dots in Figure 1) were introduced to the model to capture curvature, with values of −α and +α. We employed the Central Composite Circumscribed (CCC) methodology with an α value of 1.682 ($\sqrt[{4}]{2^{n}}$, with n as the number of factors (3), being substrate protein, fat, and carbohydrate content). Table 3 displays the actual values corresponding to the theoretical points for each macronutrient content.

This approach allows for a comprehensive exploration of the effects of various substrate compositions on larval growth, contributing to our understanding of optimal feeding conditions for BSF larvae.

#### 2.1.3. Preparation of Feed Substrates for Artificial Substrates

Table 4 shows both the macronutrient composition, and the formulation used to create the substrates, as used in the artificial substrate experiment. Conditions 1–33 were tested in 5-fold and condition 34 (the centrepoint) was tested in 10-fold, resulting in a total of 175 experimental units.

#### 2.1.4. Validation of the Experimental Design

For the validation of the model, a mix of organic side-streams was used. A recent study focused on the growth of *H. illucens* on 12 organic side-streams, which were chemically characterised, as presented in Table 5 [11]. The substrates consisted of pulp (pulp left behind after apple-juice production), beer draff (side-stream from beer-brewing), industrial food waste (supermarket and restaurant waste), chicken manure (mix of wood-pulp bedding and chicken manure), corn meal (ground corn), forced chicory roots (matured roots of chicory), fruit puree (fruit overproduction, mixed into a slurry), grain middlings (side-stream from wheat-industry), household food waste (organic food waste picked up from household containers, mixed into a slurry), hydrolysed feather meal (ground-up feathers from the chicken-industry, that underwent hydrolysation), and vegetable overproduction (overproduction from auctions, mixed into a slurry). The side-streams were selected on the basis of their availability in the Flanders region, Belgium. The side-streams were ground (Robot Coupe blixer 23, robot-coupe, Utrecht, The Netherlands) and frozen at −20 °C until used [11].

**Figure 2:**
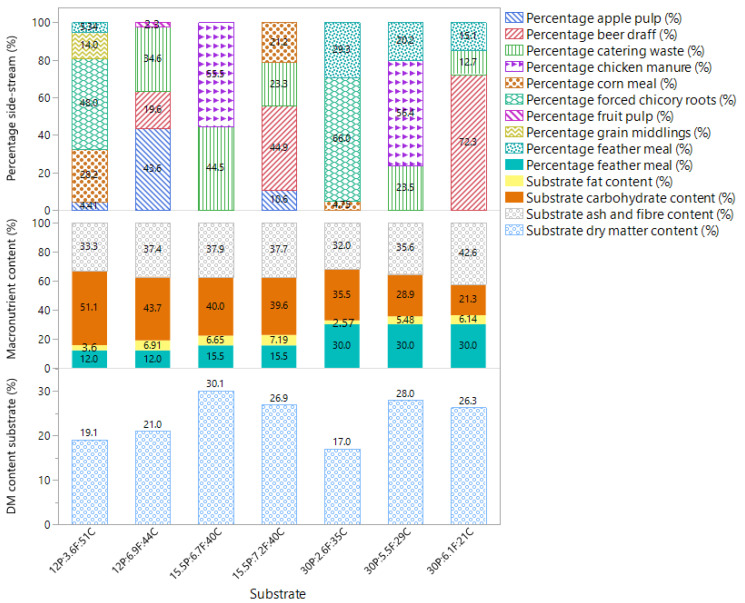
Composition of substrate mixtures used for the validation experiment. The top part displays the percentage of each side-stream used in the mix (based on dry matter), the middle part displays the macronutrient composition of the substrate (based on dry matter), and the bottom part displays the dry matter content (%).

For the validation experiment, the substrate mixtures were composed as presented in Figure 2. Substrates were composed of 12%, 15.5%, and 30% crude protein content, allowing maximising inclusion of the different side-streams.

### 2.2. Black Soldier Fly Rearing and Maintenance

BSF larvae are continuously maintained by the Centre of Expertise in Sustainable Biomass and Chemistry at Thomas More University of Applied Sciences, Belgium at the Insect Pilot Plant. An egg quantity of 1 g was harvested (eggs deposited over the course of 48 h) and collected in a plastic weighing dish. This dish was placed upon a mixture of 100 g chicken feed (Chicken Start Mash 259, AVEVE, Belgium) and 100 mL tap water (total dry matter content of 45%). The container was incubated in a climate chamber at 27 ± 1 °C at 60% RH (relative humidity). On day 3 after collecting the eggs, the weighing dish was removed and the substrate including neonates was gently mixed using a table spoon. At day 4, the young larvae including substrate/frass mixture were transferred to a larger container containing 240 g of chicken feed and 360 g of water. For this experiment, 8-day-old larvae, calculated as days after harvest, were used at the start of the experiment (day 0).

### 2.3. Experimental Setup

**For the optimisation of substrate dry matter content**, 500 larvae (mean larval mass of 3–6 mg) were counted and separated from the nursing container using tweezers. In triplicate, plastic containers (17.5 × 11.9 × 5.9 cm), closed with a mesh lid, were filled with 100 g dry matter of artificial substrate to which the appropriate amount of water was added (targeting 30% DM or maximal WHC for artificial substrates) and 500 larvae were added to each of these containers. The amount of added substrate corresponds to 0.2 g DM per larva. A sheet of aluminium foil was placed on the substrate directly contacting approximately 80% of the substrate surface area, to prevent moisture loss through evaporation. At day 9 of the experiment, the larvae were separated from their frass, counted, weighed, and dry matter content was determined, by oven drying at 105 °C for 24 h.

**For the development of artificial substrates and prediction models,** 100 larvae (3–6 mg) were counted and separated from the nursing container using tweezers. In five-fold, plastic containers (6 cm diameter) were filled with 20 g dry matter of artificial substrate to which the appropriate amount of water was added (maximal WHC). To each of these containers, 100 larvae were added. The amount of added substrate corresponds to 0.2 g DM per larva. Also, a sheet of aluminium foil was placed on the substrate directly contacting approximately 80% of the substrate surface area, to prevent moisture loss through evaporation. At day 9 of the experiment, the larvae were separated from their frass, larvae were counted, weighed, and dry matter content was determined. Additionally, the total mass and dry matter content of the residues was determined. Larval survival was determined by counting larvae at the start and end of the feed experiments. At the end of the experiment, the total larval yield and residue were measured, and subsequently, the determination of dry matter content was performed (by oven drying at 105 °C for 24 h). This sequential approach allowed the calculation of larval growth parameters, such as bioconversion efficiency.

**For the validation of the experimental design,** 500 larvae (3–6 mg) were counted and separated from the nursing container using tweezers. In triplicate, plastic containers (17.5 × 11.9 × 5.9 cm) were filled with 100 g dry matter of various side-streams-mixes, and brought to maximal water holding capacity. The amount of added substrate corresponds to 0.2 g DM per larva. Again, aluminium foil was added on top of the substrate to prevent moisture loss through evaporation. At day 9 of the experiment, the larvae were separated from their frass, larvae were counted, weighed, and dry matter content was determined. Additionally, the total mass and dry matter content of the residues was determined. Larval survival was determined by counting larvae at the start and end of the feed experiments. At the end of the experiment, the total larval yield and residue were measured, and subsequently, the determination of dry matter content was performed (by oven drying at 105 °C for 24 h). This sequential approach allowed the calculation of larval growth parameters, such as bioconversion efficiency.

### 2.4. Calculations

The calculations displayed below were executed according to Broeckx et al., 2021 [11]. The larval survival rates were determined through the division of the larval count at the end of the experiment (day 9) by the initial larval population count at the start of the experiment.

(2) $$Larval\;survival\;rate\left(\%\right)=\frac{Number\;of\;larvae\;at\;end\;of\;experiment}{Number\;of\;larvae\;at\;start\;of\;experiment}\times \;100$$

Bioconversion efficiency (BE) was calculated as following:

(3) $$BE\;\left(\%\right)=\frac{Lend,dry\;matter-Lstart,dry\;matter}{Ddry\;matter}\;\times \;100$$

with L_end_ being the larval biomass at the end of the experiment and L_start_ being the larval biomass at the start of the experiment (both expressed in dry matter). D corresponds to the amount of added substrate (expressed in g dry matter).

### 2.5. Statistical Analysis 

All statistical analysis and graphical illustrations were drafted using the JMP Pro17.0.0 software package from SAS (Buckinghamshire, UK). Data were tested for normality using a Shapiro–Wilk′s test and Levene′s test to examine the homogeneity of variance.

To determine significant differences in larval survival ratios, a *t*-test was applied with a significance threshold of *p* = 0.05. The prediction model was constructed in JMP. Least square linear regression was performed to investigate the relation between substrate macronutrient contents (independent variable) and bioconversion efficiency (dependent parameters). A full factorial approach was applied, including both main and interaction effects of the factors. Additionally, the quadratic effects of the factors (e.g., protein content^2^) were incorporated into the model.

## 3. Results

### 3.1. Optimisation of Substrate Dry Matter Content

In this experiment, a fractional factorial design was tested using 32 conditions (Table 2), with substrates hydrated to both 30% DM and to maximal WHC. During the process of substrate production, it was observed that achieving a uniform 30% DM content led to vast differences in visual appearance of the substrates related to moisture content. Certain substrates, depending on their composition, exhibited characteristics similar to dry powders, while others clearly contained free water. These visual appearances correlated with the documented maximal WHCs, i.e., a “visually wet substrate” occurs when the %DM at maximal WHC is higher than 30%. In contrast, when substrates were all hydrated according to their maximal WHC, no visually apparent differences are noted in dryness of wetness between the different substrates.

**Figure 3:**
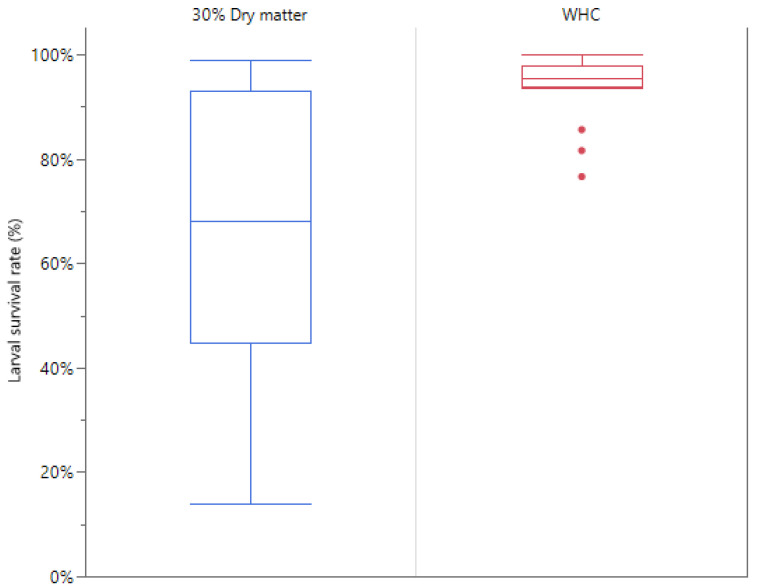
Boxplot of larval survival rates after feeding on substrates brought to 30% dry matter content (**left**) and to maximal water holding capacity (**right**).

After the rearing experiment, the influence of the suboptimality of 30% DM contents was also visible when comparing the survival rates between the two conditions. As can be observed in Figure 3, the larval survival rates are significantly higher for substrates brought at maximal water holding capacity compared to substrates with a fixed 30% dry matter content (*p* = 0.0006).

### 3.2. Development of Artificial Substrates and Prediction Models

After separation of the larvae from the frass, weighing the fresh larvae, weighing the frass, counting the larvae, and determining the frass and larval dry matter content, a dataset cleanup was performed. All data with clear measuring mistakes during the set-up/harvest (e.g., tipped over drying containers, wrongly weighed substrate ingredients) were removed (n = 6), data points showing survival ratios of less than 80% were excluded (n = 2), and finally clear outliers with significantly lower bioconversion efficiencies than same conditions (*p* < 0.05) were excluded from further analyses (n = 21). In total, the dataset was reduced from 175 to 146 data points. For the analyses, at least 3 out of 5 replicates were still present for each test condition and for the centrepoint, 8 of the 10 replicates remained in the design.

**Figure 4:**
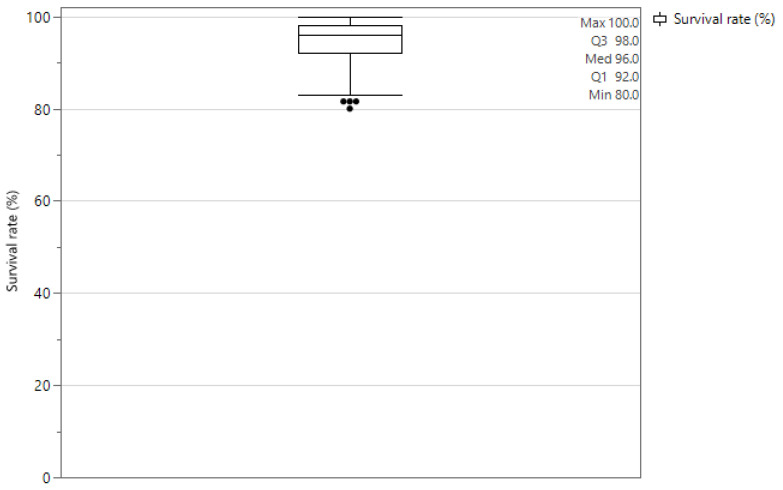
Bar-plot displaying larval survival rates in experimental set-up. The lowest point shows 80% survival (n = 146).

In Figure 4, the larval survival ratios over the course of the experiment are displayed through a boxplot. Since larval density plays a crucial role in feed conversion efficiency, larval weight, and waste reduction, it is important to maintain high and relatively consistent survival ratios. Therefore, only conditions with survival ratios of over 80% are included for further data analysis. The results of the growth experiment are displayed in Table 6.

The cleaned dataset was then used to draft a model to describe the effects of protein, fat, carbohydrate contents and the interaction effects thereof. Bioconversion efficiency was set as response. All main, interaction, and quadratic effects were significant, as shown in Table 7, which could be used to also generate a prediction formula.

The model has an R^2^ of 0.68 and *p* < 0.0001 describing a significant effect of substrate carbohydrate content (*p* < 0.0001), fat content (*p* = 0.0008), protein content (*p* = 0.0121), and the interaction effects thereof, as well as the quadratic effects. The parameter estimates are displayed in Table 7, with their respective *p*-values and parameter estimates.

The formula predicted by the model to predict larval growth is as follows:

Bioconversion efficiency (%) = 16.8503 − 0.03304 × C − 0.2531 × F − 0.0132 × (C − 36.4177) × (F − 5.9561) + 0.05681 × P − 0.0094 × (C − 36.4177) × (P − 17.4877) − 0.0404 × (F − 5.9561) × (P − 17.4877) + 0.0015 × (C − 36.4177) × (F − 5.9561) × (P − 17.4877) − 0.0024 × (C − 36.4177)^2^ − 0.0340 × (F − 5.9561)^2^ − 0.0387 × (P –17.4877)^2^ (4)

with

P = Substrate protein content (%);

F = Substrate fat content (%);

C = Substrate carbohydrate content (%).

### 3.3. Validation of the Experimental Design

To validate the experimental design, we mixed different side-streams to create substrates with varying crude protein contents: 12%, 15.5%, and 30%. Feather meal was used as a primary protein source in some substrates, though preliminary tests indicated it was indigestible for the larvae. Consequently, the protein content derived from feather meal was calculated and an adjusted total protein content was calculated for each condition (Table 8).

**Figure 5:**
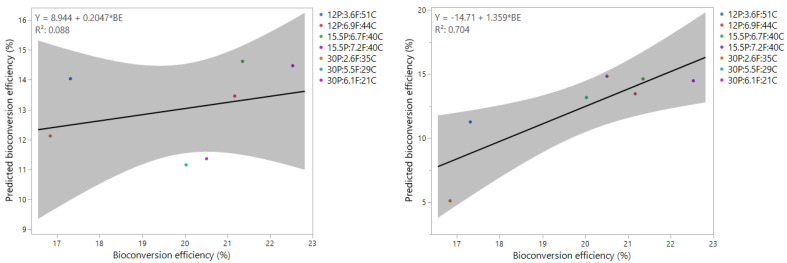
Correlation between predicted and observed bioconversion efficiency (%) with the bioconversion prediction based on raw macronutrient composition (**left**) and with the prediction of bioconversion efficiency corrected for feather meal protein (**right**).

The bioconversion efficiency was determined based on both the initial macronutrient composition and the adjusted composition accounting for the indigestible protein from feather meal. Without correcting for feather meal, no significant correlation was observed between the measured and predicted bioconversion efficiencies. However, when corrections were made for the feather meal content, a linear correlation emerged (R^2^ = 0.704). This adjustment underscores the importance of considering protein source digestibility in optimising substrate formulations for BSFL growth (Figure 5).

## 4. Discussion

### 4.1. Optimisation of Substrate Dry Matter Content

For the optimisation of substrate dry matter content, the data clearly showed that 30% dry matter content was not optimal, as larval survival ratios showed high variability. In these cases, larval density changes drastically, which has been shown to impact bioconversion efficiency [30]. By hydrating the substrates to their substrate-specific maximal WHC, significantly higher larval survival ratios were observed (*p* = 0.0006). Also, by the end of the experiment, the substrates were still damp and suitable for larval growth, in contrast to many of the substrates at 30% DM. Frooninckx et al. showed that substrates at their WHC show the most optimal bioconversion efficiency for BSFL growth; thus, WHC was used for the development of artificial substrates and prediction models [30]. Based on the data of larvae grown on substrates at full WHC from the optimisation of substrate dry matter content, an attempt was made to build a prediction model, but the number of data points was too low to do this.

### 4.2. Development of Artificial Substrates and Prediction Models

Prior to the experiment on prediction modelling through artificial substrates displayed in this paper, we tested central composite designs to determine the effects of substrate fat, carbohydrate, and protein contents on bioconversion efficiency. However, first they were set up using potato starch. The model predicted no significant impact of substrate carbohydrate contents on larval growth, which was in contrast to the literature [23]. Also, the model predicted very high substrate protein and fat contents to be most optimal for larval growth. Therefore, it was hypothesised that larvae were not digesting raw potato starch well and that it was more considered as a fibre opposed to being a carbohydrate. This was confirmed in several studies [33,34]. Therefore, the experiment was completely repeated with corn starch. Again, no significant effects of carbohydrates on larval growth were observed. Raw corn starch seemed to be poorly digestible for the larvae too, as confirmed by Guillaume et al., 2023 [33]. The same study showed that wheat starch was well-digested, which is the reason the experiment was repeated with wheat starch afterwards. The results of the trials with potato and corn starch are not elaborated on in this publication.

This model built through artificial substrates predicted highest larval final dry weight at a protein content of 23.48%, a substrate carbohydrate content of 20.64%, and a fat content of 1.91%, based on dry matter. Below, a simulation table is displayed which, based on the prediction formula, calculated the most optimal crude protein contents and the corresponding predicted bioconversion efficiencies (with 95% confidence intervals) at crude fat contents ranging from 2.00 to 8.00% and carbohydrate contents (CHCs) ranging from 10.00 to 60.00% (Table 9).

It can be observed that substrates with low CF and CHC require a higher CP content to maximise bioconversion efficiencies. Based on these set ranges (2.00–8.00% CF and 10.00–60.00% CHC), most optimal CP contents for maximising the bioconversion efficiency ranged between 15.23 and 25.50%.

**Figure 6:**
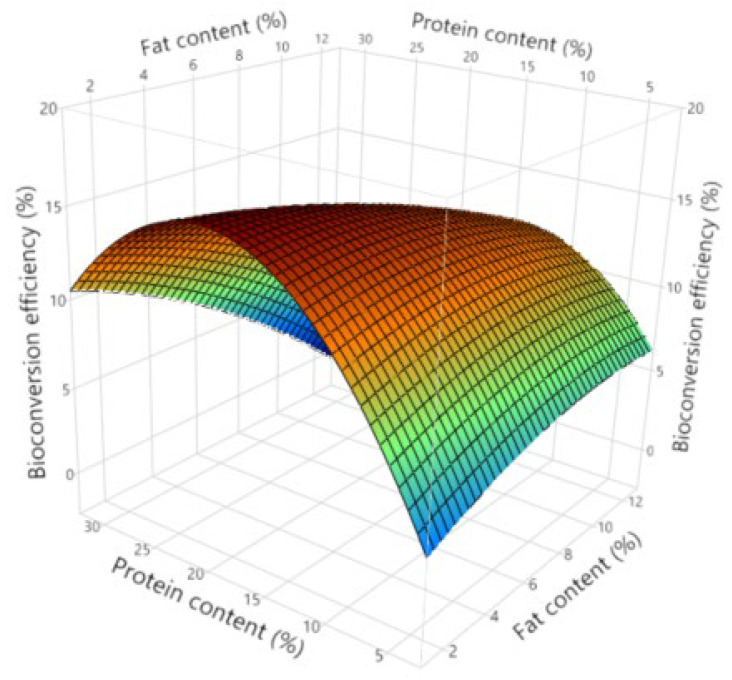
Surface plot describing interaction effects between substrate fat and protein contents on bioconversion efficiency.

In Figure 6, a surface plot is displayed showing the interaction effects of substrate protein and fat contents on bioconversion efficiency. It can be observed that bioconversion efficiency is maximised at approximately 23% substrate protein content and at 2% substrate fat content. At higher fat concentrations (8%), the most optimal protein content is approximately 17.5%; however, the highest bioconversion efficiencies are present at low substrate fat contents and approximately 23% substrate protein contents. At high protein contents, lower fat contents are desirable, while at low substrate protein contents, higher fat contents are desirable.

**Figure 7:**
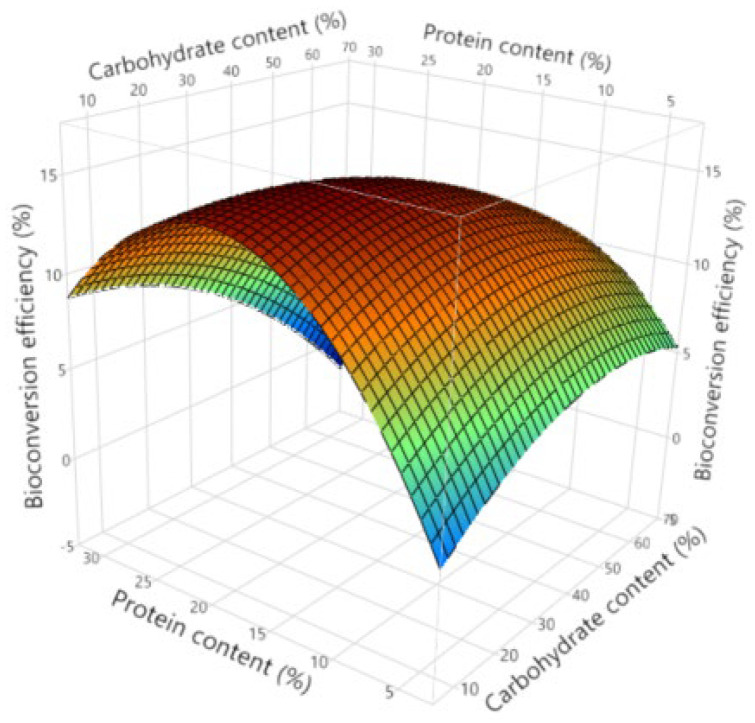
Surface plot describing interaction effects between substrate carbohydrate and protein contents on bioconversion efficiency.

In Figure 7, the interaction effects between substrate protein and carbohydrate contents are displayed. As can be observed by studying the surface plot, in general the carbohydrate content has a positive effect on the bioconversion efficiency, unless it is combined with high substrate protein contents. At low carbohydrate contents (e.g., 10%), the most optimal bioconversion efficiency is observed at approximately 20% protein. At high carbohydrate contents (e.g., 70%) the most optimal substrate protein content is approximately 15%. The most optimal combination in general for highest bioconversion efficiencies is around 20% of carbohydrates and 22% of protein.

**Figure 8:**
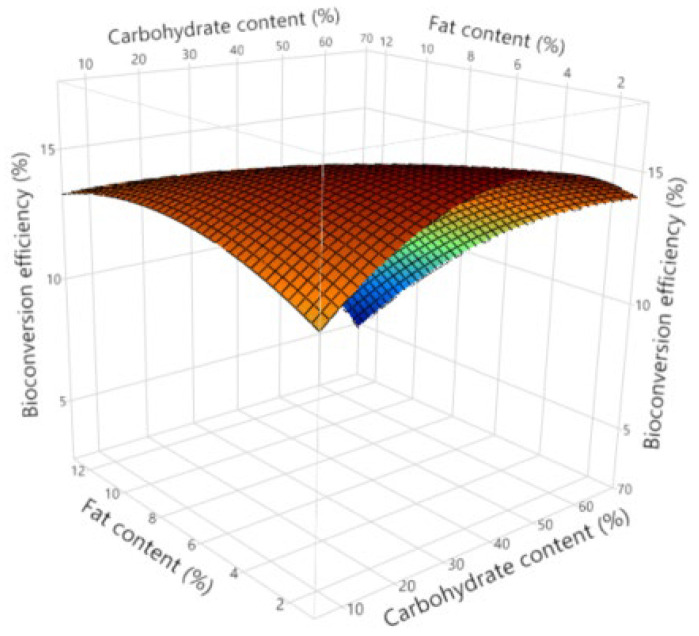
Surface plot describing interaction effects between substrate fat and carbohydrate contents on bioconversion efficiency.

In Figure 8, the interaction effects between substrate fat and carbohydrate content on bioconversion are displayed. It is observed that increased substrate fat content shows a positive effect on bioconversion efficiency when substrate carbohydrate contents are low, but an adverse effect is present when substrate carbohydrate contents are high.

In summary, substrate protein content has the most significant impact on bioconversion efficiency. This is logical, as proteins are essential for the larvae to build larval protein tissues, such as muscles. Insufficient protein leads to stunted growth [35]. When the substrate has low fat and carbohydrate content, larvae require high protein levels to achieve maximum bioconversion efficiency. This is likely because a higher proportion of substrate protein is used for energy (e.g., glucogenic amino acids used as energy source) rather than tissue building [36]. Additionally, high protein breakdown necessitates increased nitrogen release through uric acid, an energy-consuming process [37], which may explain the adverse effects observed when substrate protein content exceeds approximately 25%. Moreover, excessive protein concentrations could even lead to protein poisoning, similar to mammals [38].

Therefore, optimising larval growth requires balancing substrate fat and carbohydrates. Although high substrate fat content generally negatively impacts larval growth, it can significantly enhance bioconversion efficiency when substrate protein and carbohydrates are low. For instance, at 12% CP and 10% CHC, 13.8% CF is optimal, increasing bioconversion efficiency from 8.88% to 13.61%, as predicted by the model. Conversely, when substrate carbohydrate and protein contents are already high, high fat contents negatively influences bioconversion efficiency. This could be due to less efficient fat uptake in larvae compared to carbohydrate uptake, fat displacing water in a wet feed and limiting larval water uptake, or high fat content negatively affecting microbial communities within the larval gut, similar to mammals [39].

Similarly, higher carbohydrate contents positively affect bioconversion efficiency when substrate protein content is low. However, exceeding a carbohydrate content of approximately 50% invariably results in lower bioconversion efficiency. This could be due to metabolic overload where excessive carbohydrates might overwhelm larval metabolic processes [40,41], due to osmotic stress which may impair nutrient absorption [42], or due to microbial competition, as high carbohydrate contents may promote the growth of certain microbial populations that compete with larvae for nutrients [43]. In this study, cellulose powder was used as a fibre to use as a filler to complete the substrates. Even though recent studies highlighted significant effects of fibres on larval growth [44], during this study, no significant effects of cellulose content on larval growth were observed.

### 4.3. Validation of the Experimental Design

Although the validation experiment demonstrated a linear correlation with an R^2^ of 0.704, a 1-to-1 ratio between predicted and measured bioconversion efficiency was not observed. Several factors influence this outcome, including larval genetics [45], larval microbiome [43], larval handling stress [46], larval age at harvest [11], larval density [47,48], the total volume of the containers [48], and the evaporation rate of moisture during the experiment [44]. Despite the absence of a 1-to-1 ratio, it is anticipated that similar trends will be present in real-life substrates within the defined macronutrient boundaries of this model. Factors such as macronutrient digestibility and completeness of for example amino-acid profiles will also play a significant role (see further).

Validation of the experimental design using real side-streams is extremely challenging due to several factors, primarily related to protein content. Since protein content is a major contributor to bioconversion efficiency, issues in this area can significantly impact the model’s effectiveness. Proteins vary in their amino acid compositions and digestibilities, which can be assessed using the Protein Digestibility Corrected Amino Acid Score (PDCAAS) [49]. PDCAAS ranges from 0 (lowest) to 1 (highest), decreasing when a protein is deficient in essential amino acids or has low digestibility [49]. Amino acids from complete proteins can fully be utilised for larval protein synthesis, while amino acids coming from incomplete proteins are proportionally more used for processes such as energy production (e.g., glucogenic amino acids) [36]. Unlike in humans, the optimal amino acid profile for larvae and their protein digestibility are largely unknown. Additionally, protein digestibility is often influenced by the degree of substrate processing (e.g., denaturation) [50]. In insect rearing, particularly with manure substrates, high amounts of non-protein nitrogen (NPN) are present [51], and the metabolic pathways insects use to process it are not fully understood. High microbial load substrates, common in black soldier fly rearing [52], add another layer of complexity. Microbiota can convert non-protein nitrogen into protein [51], degrade specific matrices like cellulose (which can incapsulate protein that otherwise would be indigestible) [53], consume substrate proteins [43], or transform substrate protein into microbial protein [54]. These processes complicate the direct use of protein content measurements. To address these challenges, future studies should implement correction factors based on the protein source used, ensuring more accurate assessments of bioconversion efficiency.

For substrate fat contents, the fatty acid composition is probably less crucial than for the protein. However, a balanced fatty acid profile with essential fatty acids is important. Moreover, certain fatty acids serve as precursors to hormones and signalling molecules (e.g., eicosanoids from arachidonic acid), which can influence growth rates, immune responses, and overall metabolism [55]. Also, some fatty acids can regulate metabolism related gene expression, as some downregulate or upregulate genes involved in lipid metabolism, protein synthesis, and growth factor production, as already explored in humans [56]. This study also focused more on the lower substrate fat contents (2.00–12.73%), as the aim was bioconversion optimisation rather than testing all ranges. This means the model is only accurate for substrate fat contents within these ranges. Therefore, some substrates, such as slaughterhouse and hatchery wastes, cannot be used in this model [22,57].

The carbohydrate content of substrates is significantly influenced by the specific types of carbohydrates present. The primary non-fibre carbohydrate sources in side-streams include (1) simple sugars (monosaccharides and disaccharides), (2) oligosaccharides, (3) polysaccharides (such as starch, hemicellulose, and pectin), and (4) other complex carbohydrates like xylans and beta-glucans [58]. Each of these carbohydrate categories follows distinct metabolic pathways, and even within subcategories, such as different types of starch, there are considerable differences in digestibility [59]. These differences are attributed to factors like the amylose to amylopectin ratio, granule structure and size, and crystallinity [60]. Additionally, processing steps such as gelatinisation and retrogradation significantly impact digestibility [33,61]. Furthermore, dietary fibres can form physical barriers around starch, hindering enzyme access, and protein–starch or lipid–starch complexes can form, further reducing digestibility [62]. Consequently, similar to protein sources, correction factors should be applied based on the specific type of carbohydrate source used.

## 5. Conclusions

The optimisation of substrate dry matter content for BSFL growth revealed that maintaining substrates at their maximal WHC is crucial for maximising larval survival rates and bioconversion efficiency. Substrates with 30% dry matter content resulted in inconsistent larval survival, highlighting the necessity of adequate hydration. Model predictions indicated the highest larval final dry weight at a protein content of 23.48%, carbohydrate content of 20.64%, and fat content of 1.91%, based on dry matter. For practical application, an Excel-based tool to optimise substrate macronutrient compositions using the prediction model developed in this publication is available as Supplementary Materials. Simulations demonstrated that substrates with low fat and carbohydrate contents required higher protein levels to maximise bioconversion efficiency. Optimal crude protein contents for maximising efficiency ranged between 15.23% and 25.50% within the tested fat and carbohydrate ranges. In validating these models with real side-streams, challenges were noted, particularly regarding protein content due to variations in amino acid composition and digestibility. Correction factors should be applied based on the specific protein and carbohydrate sources used to enhance model accuracy. Future research should focus on refining these models further by incorporating these correction factors and expanding the range of substrate components tested. This would lead to more accurate predictions of bioconversion efficiency, contributing to more efficient and sustainable practices in BSFL rearing.

## Figures and Tables

**Table 1:** Dry matter contents and macronutrient compositions of feed ingredients used for composing artificial substrates.

	Chickens Start Mash	Sunflower Oil	Wheat Starch	Casein	Cellulose
Dry matter (%)	89%	100%	88%	92%	94%
Protein (% DM)	20%	0%	0%	78%	0%
Fat (% DM)	5%	100%	0%	2%	0%
CHC (% DM)	48%	0%	91%	11%	0%

CHC (%): carbohydrate content (%).

**Table 2:** Macronutrient composition and dry matter content of substrates during the optimisation of substrate dry matter contents.

Dry Matter Content Fixed to 30%					Dry Matter Content Based on Maximal WHC				
Nr.	Protein (%)	Fat (%)	CHC (%)	Dry Matter (%)	Nr.	Protein (%)	Fat (%)	CHC (%)	Dry Matter (%)
1	10	15	55	30%	**17**	10	15	55	46%
2	25	2	55	30%	**18**	25	2	55	41%
3	25	2	55	30%	**19**	25	2	55	41%
4	10	2	10	30%	**20**	10	2	10	29%
5	10	2	55	30%	**21**	10	2	55	38%
6	25	15	10	30%	**22**	25	15	10	38%
7	10	15	10	30%	**23**	10	15	10	32%
8	10	15	55	30%	**24**	10	15	55	46%
9	25	2	10	30%	**25**	25	2	10	32%
10	25	15	55	30%	**26**	25	15	55	50%
11	25	15	10	30%	**27**	25	15	10	38%
12	25	15	55	30%	**28**	25	15	55	50%
13	25	2	10	30%	**29**	25	2	10	32%
14	10	2	55	30%	**30**	10	2	55	38%
15	10	2	10	30%	**31**	10	2	10	29%
16	17.5	8.5	32.5	30%	**32**	17.5	8.5	32.5	39%

CHC (%): carbohydrate content (%); nr.: substrate identification number.

**Table 3:** Macronutrient boundaries, categorised as (−α, −1, 0, +1, +α), wherein −1 represented the lower boundary, +1 the upper boundary, 0 the midpoint, and α the axial points with the corresponding substrate protein, fat, and carbohydrate contents.

Macronutrient Composition	−α	−1	0	1	+α
Substrate protein content (%)	4.89	10	17.5	25	30.12
Substrate fat content (%)	/	2	6	10	12.73
Substrate carbohydrate content (%)	8.07	20	37.5	55	66.94

**Table 4:** Nutritional composition of different substrates used in the experiment with carbohydrate (CHC), fat, and protein contents displayed based on dry matter substrate. Dry matter contents are based on the maximal water holding capacity of substrates and the substrates are composed of chicken start mash (CSM), sunflower oil, wheat starch, casein powder, and cellulose powder. Percentages of each ingredient are displayed based on dry matter.

Chemical Composition (Dry)					Composed of (Dry)				
Nr.	CHC (%)	Fat (%)	Protein (%)	Dry Matter (%)	CSM Dry (%)	Sunflower Oil (%)	Wheat Starch (%)	Casein (%)	Cellulose (%)
1	20.00	6.00	10.00	31.90	8.01	5.38	16.49	10.77	59.34
2	55.00	2.00	10.00	39.49	8.01	1.38	55.06	10.77	24.78
3	55.00	2.00	25.00	39.51	8.01	1.00	52.73	30.00	8.26
4	55.00	10.00	25.00	45.41	8.01	9.00	52.73	30.00	0.26
5	55.00	10.00	10.00	45.38	8.01	9.38	55.06	10.77	16.78
6	20.00	10.00	10.00	33.66	8.01	9.38	16.49	10.77	55.34
7	20.00	10.00	25.00	33.68	8.01	9.00	14.16	30.00	38.83
8	20.00	2.00	25.00	30.32	8.01	1.00	14.16	30.00	46.83
9	55.00	2.00	17.50	39.50	8.01	1.19	53.89	20.38	16.52
10	37.50	2.00	10.00	34.30	8.01	1.38	35.77	10.77	44.06
11	37.50	2.00	25.00	34.31	8.01	1.00	33.44	30.00	27.55
12	20.00	2.00	17.50	30.32	8.01	1.19	15.33	20.38	55.09
13	20.00	6.00	10.00	31.90	8.01	5.38	16.49	10.77	59.34
14	55.00	6.00	10.00	42.23	8.01	5.38	55.06	10.77	20.78
15	55.00	6.00	25.00	42.26	8.01	5.00	52.73	30.00	4.26
16	20.00	6.00	25.00	31.91	8.01	5.00	14.16	30.00	42.83
17	37.50	10.00	10.00	38.65	8.01	9.38	35.77	10.77	36.06
18	20.00	10.00	17.50	33.67	8.01	9.19	15.33	20.38	47.09
19	55.00	10.00	17.50	45.39	8.01	9.19	53.89	20.38	8.52
20	37.50	10.00	25.00	38.67	8.01	9.00	33.44	30.00	19.55
21	37.50	2.00	17.50	34.31	8.01	1.19	34.61	20.38	35.80
22	37.50	2.00	17.50	34.31	8.01	1.19	34.61	20.38	35.80
23	37.50	12.72	17.50	40.41	8.01	11.92	34.61	20.38	25.08
24	66.94	6.00	17.50	46.96	8.01	5.19	67.04	20.38	0.00
25	8.07	6.00	17.50	29.45	8.01	5.19	2.18	20.38	64.24
26	37.50	6.00	30.12	36.37	8.01	4.87	32.65	36.55	17.91
27	37.50	6.00	4.89	36.34	8.01	5.52	36.57	4.21	45.69
28	37.50	10.00	17.50	38.66	8.01	9.19	34.61	20.38	27.80
29	55.00	6.00	17.50	42.24	8.01	5.19	53.89	20.38	12.52
30	37.50	6.00	10.00	36.35	8.01	5.38	35.77	10.77	40.06
31	37.50	6.00	25.00	36.36	8.01	5.00	33.44	30.00	23.55
32	37.50	2.00	17.50	34.31	8.01	1.19	34.61	20.38	35.80
33	20.00	6.00	17.50	31.91	8.01	5.19	15.33	20.38	51.09
34	37.50	6.00	17.50	36.35	8.01	5.19	34.61	20.38	31.80

CHC (%): carbohydrate content (%); nr.: substrate identification number.

**Table 5:** Proximate analysis of side-streams with dry matter content (DM), fat content of ether extract (EE), crude protein content (CP), crude ash content (CA), amylase-treated neutral detergent fibre content (aNDF), carbohydrate content (CHC), and gross energy content (GE). Values are reported as mean ± standard deviation (n = 3). See [11] for more details.

Substrate	DM ^1^	EE ^2^	CP ^2^	CA ^2^	aNDF ^2^	CHC ^2^	GE ^3^
Apple pulp	25.9 ± 0.1	4.6 ± 0.1	3.4 ± 0.0	1.9 ± 0.1	43.3 ± 1.0	47.0 ± 1.2	242
Beer draff	29.4 ± 0.5	5.5 ± 0.2	19.4 ± 0.6	4.5 ± 0.1	50.6 ± 0.4	20.0 ± 1.3	207
Catering waste	17.8 ± 0.7	10.9 ± 0.6	18.5 ± 0.1	9.2 ± 0.0	7.8 ± 0.4	53.5 ± 1.1	386
Chicken manure	67.3 ± 3.2	3.3 ± 0.1	13.1 ± 0.2	13.0 ± 0.1	41.5 ± 0.6	29.1 ± 1.0	198
Corn meal	87.0 ± 0.0	8.1 ± 0.1	9.5 ± 0.2	2.3 ± 0.1	32.8 ± 2.0	47.3 ± 2.4	300
Forced chicory roots	15.7 ± 0.2	0.9 ± 0.0	4.6 ± 0.1	31.0 ± 0.9	14.2 ± 0.5	49.3 ± 1.5	224
Fruit puree	6.2 ± 0.1	4.5 ± 0.1	10.0 ± 0.1	12.6 ± 0.2	33.3 ± 0.1	39.9 ± 0.5	239
Grain middlings	90.2 ± 0.1	2.9 ± 0.1	14.1 ± 0.1	7.9 ± 0.2	22.0 ± 0.9	53.1 ± 1.3	295
Vegetable overproduction	8.1 ± 0.2	2.0 ± 0.3	10.5 ± 0.4	15.3 ± 0.1	30.2 ± 1.1	42.0 ± 1.9	228
Hydrolysed feather meal	84.5 ± 0.5	5.4 ± 0.1	90.5 ± 0.1	1.7 ± 0.0	0.0 ± 0.0	2.4 ± 0.2	415

^1^ g/100 g fresh matter; ^2^ g/100 g dry matter; ^3^ kcal/100 g dry matter.

**Table 6:** Results of the rearing experiment on the artificial substrate, displaying larval survival ratio, fresh larval end mass, larval dry matter content, dry larval mass, and bioconversion efficiency. Values are reported as mean ± standard deviation (5 ≥ n ≥ 3; n = 8 for centrepoints *).

Condition	Survival Ratio (%)	Fresh Larval Mass (g)	Larval DM Content (%)	Dry Larval Mass (g)	Bioconversion Efficiency (%)
1	94.25 ± 3.10	7.94 ± 1.18	30.28 ± 2.84	2.39 ± 0.34	12.06 ± 0.42
2	92.20 ± 6.46	9.46 ± 1.63	28.92 ± 3.75	2.72 ± 0.52	12.07 ± 0.22
3	94.60 ± 4.98	8.58 ± 1.77	28.71 ± 4.45	2.43 ± 0.48	12.77 ± 1.01
4	96.00 ± 3.92	9.14 ± 2.54	33.08 ± 2.61	2.99 ± 0.71	9.02 ± 0.63
5	91.00 ± 1.00	9.01 ± 2.35	33.52 ± 4.31	2.95 ± 0.46	11.15 ± 0.61
6	96.00 ± 1.73	7.45 ± 1.29	29.92 ± 1.36	2.23 ± 0.37	12.21 ± 0.70
7	95.00 ± 7.07	9.99 ± 0.49	31.14 ± 0.28	3.11 ± 0.12	10.48 ± 0.53
8	94.67 ± 6.11	7.63 ± 2.34	31.15 ± 3.55	2.33 ± 0.57	16.04 ± 0.81
9	94.80 ± 7.98	9.34 ± 0.91	30.39 ± 3.26	2.84 ± 0.43	15.87 ± 1.02
10	95.00 ± 8.09	8.63 ± 1.91	29.95 ± 3.17	2.61 ± 0.78	11.20 ± 0.81
11	96.80 ± 4.09	8.78 ± 2.68	31.75 ± 1.99	2.77 ± 0.82	12.71 ± 1.05
12	96.50 ± 0.71	8.31 ± 1.79	31.92 ± 3.15	2.62 ± 0.31	13.94 ± 0.56
13	92.67 ± 6.51	9.01 ± 2.20	31.15 ± 1.59	2.79 ± 0.63	10.52 ± 1.39
14	94.20 ± 4.38	8.65 ± 1.56	30.07 ± 2.08	2.58 ± 0.37	12.30 ± 0.52
15	93.00 ± 2.45	9.35 ± 2.01	29.41 ± 2.41	2.72 ± 0.46	10.12 ± 0.76
16	96.20 ± 2.17	9.26 ± 1.02	26.98 ± 2.74	2.48 ± 0.26	16.29 ± 2.54
17	93.00 ± 6.27	8.34 ± 2.21	33.73 ± 2.33	2.78 ± 0.62	12.01 ± 1.72
18	95.75 ± 0.96	8.09 ± 2.49	34.09 ± 3.76	2.72 ± 0.66	16.47 ± 1.64
19	91.00 ± 7.57	7.69 ± 1.57	30.25 ± 1.30	2.31 ± 0.37	10.51 ± 0.64
20	91.00 ± 6.78	10.09 ± 1.66	30.41 ± 2.52	3.05 ± 0.51	9.46 ± 1.16
21	92.75 ± 7.27	7.84 ± 1.18	30.76 ± 2.74	2.39 ± 0.23	16.87 ± 1.24
22	96.50 ± 2.08	10.51 ± 1.31	29.76 ± 4.10	3.12 ± 0.52	17.26 ± 0.98
23	92.25 ± 5.19	9.08 ± 2.07	29.14 ± 2.61	2.61 ± 0.45	12.10 ± 1.06
24	96.40 ± 2.07	9.32 ± 2.56	32.15 ± 3.04	2.97 ± 0.75	11.60 ± 0.29
25	96.00 ± 1.00	10.04 ± 1.91	29.62 ± 1.67	2.98 ± 0.69	13.45 ± 0.52
26	92.80 ± 4.32	7.81 ± 1.78	32.45 ± 3.36	2.52 ± 0.53	10.64 ± 0.38
27	96.80 ± 1.92	9.76 ± 1.51	29.16 ± 1.99	2.84 ± 0.49	8.47 ± 0.31
28	94.40 ± 3.58	8.64 ± 1.87	29.28 ± 1.36	2.52 ± 0.52	10.18 ± 0.64
29	97.80 ± 1.79	9.88 ± 0.78	29.35 ± 4.07	2.88 ± 0.30	14.37 ± 0.29
30	95.80 ± 2.28	9.31 ± 1.57	31.49 ± 2.02	2.93 ± 0.57	14.24 ± 1.59
31	96.40 ± 2.19	9.13 ± 3.09	33.86 ± 3.64	3.01 ± 0.73	11.86 ± 1.09
32	97.50 ± 3.54	7.91 ± 2.42	35.04 ± 8.38	2.67 ± 0.18	16.59 ± 0.82
33	93.75 ± 5.74	10.59 ± 1.36	30.81 ± 3.31	3.23 ± 0.25	14.69 ± 0.69
34 *	93.50 ± 5.86	9.41 ± 0.75	31.91 ± 1.32	3.01 ± 0.31	14.28 ± 1.53

**Table 7:** Parameter estimates describing main, quadratic, and interaction effects of substrate protein, fat, and carbohydrate contents on bioconversion efficiency.

Term	Estimate	Std Error	t Ratio	*p*-Value
Intercept	16.850344	0.619	27.22	<0.0001
Carbohydrate content (%)	−0.033039	0.009631	−3.43	0.0008
Fat content (%)	−0.253072	0.043873	−5.77	<0.0001
(Carbohydrate content (%) − 36.4177) × (Fat content (%) − 5.95611)	−0.013192	0.003373	−3.91	0.0001
Protein content (%)	0.0568106	0.022327	2.54	0.0121
(Carbohydrate content (%) − 36.4177) × (Protein content (%) − 17.4877)	−0.009372	0.001766	−5.31	<0.0001
(Fat content (%) − 5.95611) × (Protein content (%) − 17.4877)	−0.040412	0.008059	−5.01	<0.0001
(Carbohydrate content (%) − 36.4177) × (Fat content (%) − 5.95611) × (Protein content (%) − 17.4877)	0.0014897	0.0006	2.48	0.0142
(Carbohydrate content (%) − 36.4177) × (Carbohydrate content (%) − 36.4177)	−0.002363	0.00068	−3.47	0.0007
(Fat content (%) − 5.95611) × (Fat content (%) − 5.95611)	−0.034031	0.016067	−2.12	0.0360
(Protein content (%) − 17.4877) × (Protein content (%) − 17.4877)	−0.038692	0.003781	−10.23	<0.0001

**Table 8:** Validation substrates with their measured bioconversion efficiencies, as well as the predicted bioconversion efficiencies based on the model, with and without the inclusion of proteins derived from feather meal.

Substrate	Measured Bioconversion Efficiency (%)	Predicted Bioconversion Efficiency (%)	Feather Meal Protein (%)	Adjusted Protein Content (%)	Predicted Bioconversion Efficiency Without Feather Meal Protein (%)
12P:3.6F:51C	17.31 ± 0.30	14.04	4.83	7.17	11.26
30P:2.6F:35C	16.84 ± 0.55	12.12	26.50	3.50	5.12
30P:6.1F:21C	20.51 ± 1.61	11.36	13.63	16.37	14.82
15.5P:6.7F:40C	21.36 ± 1.31	14.61	0.00	15.50	14.61
30P:5.5F:29C	20.03 ± 1.09	11.16	18.25	11.75	13.17
12P:6.9F:44C	21.17 ± 1.00	13.46	0.00	12.00	13.46
15.5P:7.2F:40C	22.53 ± 1.59	14.47	0.00	15.50	14.47

**Table 9:** Simulation table showing most optimal protein contents (CPs) at fixed crude fat (CF) and carbohydrate contents (CHC) showing the corresponding predicted mean bioconversion efficiency (BE), as well as the 95% confidence intervals (BE 95% CI).

CF (%)	CHC (%)	CP (%)	BE (%)	BE 95% CI (%)	CF (%)	CHC (%)	CP (%)	BE (%)	BE 95% CI (%)
2.00	10.00	25.50	15.93	[14.14; 17.72]	6.00	10.00	21.38	14.95	[13.99; 15.90]
2.00	20.00	23.53	16.06	[15.09; 17.04]	6.00	20.00	20.17	15.32	[14.73; 15.90]
2.00	30.00	21.55	16.02	[15.41; 16.63]	6.00	30.00	18.97	15.33	[14.70; 15.96]
2.00	40.00	19.58	15.81	[15.25; 16.37]	6.00	40.00	17.76	14.97	[14.29; 15.66]
2.00	50.00	17.61	15.43	[14.78; 16.07]	6.00	50.00	16.57	14.26	[13.62; 14.91]
2.00	60.00	15.64	14.87	[13.81; 15.93]	6.00	60.00	15.36	13.19	[13.97; 12.41]
4.00	10.00	23.44	15.41	[14.25; 16.57]	8.00	10.00	19.31	14.54	[13.56; 15.51]
4.00	20.00	21.85	15.72	[15.09; 16.34]	8.00	20.00	18.50	14.86	[14.26; 15.46]
4.00	30.00	20.26	15.75	[15.20; 16.29]	8.00	30.00	17.68	14.76	[14.15; 15.38]
4.00	40.00	18.67	15.50	[14.91; 16.08]	8.00	40.00	16.86	14.24	[13.59; 14.89]
4.00	50.00	17.09	14.97	[14.38; 15.56]	8.00	50.00	16.04	13.30	[12.68; 13.92]
4.00	60.00	15.50	14.17	[13.32; 15.01]	8.00	60.00	15.23	11.94	[11.14; 12.74]

## Data Availability

The data presented in this study are available on request from the corresponding author.

## Supplementary Materials

An Excel file is available at https://thomasmore.be/en/sustainable-biomass-and-chemistry/tool/feed-optimisation-black-soldier-fly (accessed on 29 October 2024) that can be used to optimise substrate macronutrient compositions based on the prediction model, developed in this publication.
